# Comparison of Microstructure, Texture, and Mechanical Properties of TZ61 and AZ61 Mg Alloys Processed by Differential Speed Rolling

**DOI:** 10.3390/ma15030785

**Published:** 2022-01-20

**Authors:** Kamil Majchrowicz, Bogusława Adamczyk-Cieślak, Witold Chromiński, Paweł Jóźwik, Zbigniew Pakieła

**Affiliations:** 1 Faculty of Materials Science and Engineering, Warsaw University of Technology, Wołoska 141, 02-507 Warsaw, Poland; boguslawa.cieslak@pw.edu.pl (B.A.-C.); witold.chrominski@pw.edu.pl (W.C.); zbigniew.pakiela@pw.edu.pl (Z.P.); 2 Faculty of Advanced Technologies and Chemistry, Military University of Warsaw, Kaliskiego 2, 00-908 Warsaw, Poland; pawel.jozwik@wat.edu.pl

**Keywords:** Mg-Sn-Zn alloys, AZ61 alloy, differential speed rolling, microstructure, basal texture splitting, plasticity

## Abstract

In this work, the comparison of microstructure, texture, and mechanical properties of the newly developed TZ61 (Mg-6Sn-1Zn) alloy with the commercially available AZ61 (Mg-6Al-1Zn) has been presented. Both analyzed Mg alloys were processed by conventional symmetric and asymmetric rolling (i.e., Differential Speed Rolling—DSR). The microstructure and texture were examined by EBSD and XRD, whereas the mechanical behavior was investigated by uniaxial tensile tests. DSR processing led to more effective grain refinement of both TZ61 and AZ61 sheets. However, a high fraction of Mg_2_Sn phase precipitates in the TZ61 sheets hindered grain growth what resulted in their smaller grain size as compared to AZ61 sheets. DSR processing lowered also the basal texture intensity in the TZ61 and AZ61 sheets. A unique basal poles splitting was observed for the as-rolled TZ61 alloy, while AZ61 alloy exhibited a typical single-peak basal texture. Finally, the reduced grain size and weakened basal texture by DSR processing caused increase of plasticity of the annealed TZ61 and AZ61 sheets. Nevertheless, the annealed AZ61 sheets showed higher uniform elongation and strength (as compared to TZ61 ones), which has been attributed to their significantly lower texture intensity and greater ability to strain hardening.

## 1. Introduction

Wrought Mg alloys still show a poor formability and low thermal stability, which are the main reasons of their limited usage in automotive and aerospace industries [1,2,3]. Mg deformation at room temperature occurs mainly by a dislocation slip on the densely packed basal {0001} planes or {10 − 12} <10 − 11> extension twinning. Thus, additional slip systems (prismatic and pyramidal slips) have to be activated to increase their formability [4,5].

In the recent years, the density functional theory (DFT) has been adopted to calculate the so-called generalized stacking fault energy (GSFE), which shows the tendency to emission of partial dislocation and easiness of dislocation movement in the specified slip systems [6,7]. The latest studies by Muzyk et al. [7], Wang et al. [8], and Zhang et al. [9] showed that Sn strongly reduces the GSFE of Mg in different slip systems, i.e., basal {0001}<1120>, prismatic {1 − 100}<−1 − 120>, as well as pyramidal {10 − 11}<11 − 20> and {11 − 22}<11 − 23> slip modes. Thus, Sn addition to Mg alloys may activate those slip systems that are normally unfavored and lead to their formability improvement.

The positive effect of Sn on stretch formability of Mg alloys has been recently proved by Suh et al. [10]. The 1 wt.% addition of Sn instead of Zn to Mg-3Al alloy facilitated the activation of prismatic <a> slip and improved its uniform deformation and formability. Yoon and Park [11] also noted that Mg-Sn-Al-Zn alloys (TAZ541, TAZ711, TAZ811) exhibit superior forgeability at elevated forming temperature in comparison to conventional Mg-Al-Zn alloys (AZ61, AZ80). What is more important, Mg-Sn-based alloys show a great potential for high-temperature applications due to the formation of Mg_2_Sn precipitates, which are more thermally stable (melting temperature T_m_ of Mg_2_Sn is around 770 °C) than Mg_17_Al_12_ precipitates in the most common Mg-Al-Zn alloys (T_m_ = 462 °C) [12]. Mg-Sn-based alloys are an age-hardenable group of Mg alloys, but the strengthening effect by Mg_2_Sn phase precipitates is not so effective as in the case of precipitation-strengthened Al alloys [13,14]. One of the approaches to increase the strength of heat-treatable Mg-Sn-based alloys is to accelerate kinetics of Mg_2_Sn precipitates formation by microalloying, e.g., Zn, Na, or Ag [14,15,16,17].

It has been shown that the Mg-Sn-based alloys seem to be a promising group of Mg alloys. Thus, the aim of the present work is to compare the microstructure, texture, and mechanical properties of a newly developed TZ61 (Mg-6Sn-1Zn) alloy with a commercially available AZ61 (Mg-6Al-1Zn). Both analyzed Mg alloys were processed by conventional symmetric as well as asymmetric rolling. It is known that conventional symmetric rolling creates a strong basal texture, which limits the formability of sheets at a further processing step [2,4]. The improvement of their formability can be obtained by a reduction of texture intensity due to intense shear deformation [5,6]. Differential speed rolling (DSR) as an asymmetric rolling method is used in this study to modify the texture of Mg sheets. It enables for introduction of intense shear deformation by differentiation of lower and upper rolls speed [18,19,20]. DSR-processed Mg sheets exhibit enhanced ductility and formability as compared to conventionally rolled ones, as has been shown for Mg-Al-Zn [20,21,22,23], Mg-Zn-Zr [24], or Mg-Al-Mn [25] alloys. The improvement of ductility is achieved mainly by a weakening of basal texture intensity and its spreading, which allow for easier activation of basal <a> dislocation slip and {10 − 12}<10 − 11> extension twinning [20,21]. The basal texture modification is obtained even at a low speed asymmetry (the ratio between upper and lower rolls R < 1.5) [20,21,22,23]. A higher speed asymmetry (R > 2) results also in a significant grain refinement, which allows for further increase of formability [23,26]. Similar findings have been found in our previous work [27] dedicated to Mg-6Sn (wt.%) alloy processed at different speed ratios (ranging from 1 to 3). Up to now, Mg-Sn-Zn alloys processed by DSR method have been studied only by Verma et al. [28]. Thus, it is of great importance of this study to investigate more deeply the effect of shear deformation on the evolution of microstructure, texture, and mechanical properties in the analyzed TZ61 alloy and compare it to the commercially available AZ61 alloy.

## 2. Materials and Methods

### 2.1. Material Preparation

A commercially available AZ61 (Mg-6Al-1Zn) alloy as well as a newly developed TZ61 (Mg-6Sn-1Zn) alloy used in this study were received in the form of φ25 mm hot extruded rods. The chemical composition of TZ61 alloy measured by atomic absorption spectrometry was 5.95 wt.% of Sn and 1.34 wt.% of Zn. Before rolling process, the rods were machined into rectangular billets with dimensions of 200 × 20 × 10 mm and solution heat treated at 480 °C for 1 h followed by water quenching. The billets were subjected to 4 passes with a reduction ratio of 15% per pass. Before each pass, the rolled samples were heated at 400 °C for 10 min and rotated by 180° around the rolling direction. Ko and Hamad [26] showed that such DSR rolling route enables the obtaining of Mg alloy sheets with a homogeneous microstructure and a weak basal texture. TZ61 and AZ61 alloys were subjected to conventional symmetric rolling with equal speed of rolls (R = 1) as well as asymmetric rolling at a speed ratio of R = 3 (the ratio between upper and lower rolls) with the constant velocity of the upper roll maintained at 4 m/min. As shown in our previous work [27], such a relatively high speed ratio causes a significant grain refinement, texture weakening, and improvement of mechanical properties of Mg-Sn alloys. Finally, TZ61 and AZ61 sheets were investigated in the as-rolled state and after further annealing at 300 °C for 1 h.

### 2.2. Microstructure Characterization

The microstructure of as-rolled sheets was characterized by electron backscattered diffraction (EBSD) method using Hitachi (Tokyo, Japan) SU-70 scanning electron microscope (SEM) equipped with a HKL Nordlys detector. The samples for EBSD measurements were prepared by grinding and argon ion polishing at 6 kV using Hitachi (Tokyo, Japan) IM4000 Ion Milling System. EBSD maps were taken on the rolling direction-normal direction (RD-ND) plane with an acceleration voltage of 20 kV from an area of at least 100,000 μm^2^ using a step size of 0.4 μm. The collected data was analyzed by dedicated HKL Channel 5 software. The equivalent diameter (a diameter of a circle with the same area as the grain under consideration) was used to express the grain size, while the low angle grain boundaries (LAGBs, marked as faint grey lines on EBSD maps) and high angles grain boundaries (HAGBs, marked as black lines) were considered as those with the misorientation of 3 to 15° and above 15°, respectively.

The microstructure observations of annealed sheets were also conducted on the RD-ND plane using a Zeiss (Oberkochen, Germany) Axio Observer light microscope (LM). Metallographic samples were prepared by polishing using 3 and 1 μm ethanol-based diamond suspensions and etching with a solution of 2% nital. LM images were used for calculation of the equivalent diameter of grains using the MicroMeter software (v.086b) [29,30].

### 2.3. Texture and Phase Composition Analysis

The texture of as-rolled and annealed sheets was analyzed by X-ray diffraction (XRD) using Bruker (Billerica, MA, USA) D8 Discover diffractometer with Co Kα radiation (λ = 1.79 Å). Diffraction patterns were collected from the rolling direction-transverse direction (RD-TD) plane with a step size of 0.02° and scan time of 5 s per interval. The orientation distribution functions (ODFs) and complete pole figures were calculated using LaboTex software (version 3.0) based on four incomplete pole figures, i.e., (0002), (10 − 10), (10 − 11), and (10 − 12). XRD measurements were also used for the estimation of Mg_2_Sn phase volume fraction calculated by the Reference Intensity Ratio (RIR) method [31] using the Match! software.

### 2.4. Mechanical Properties Characterization

The mechanical properties of TZ61 and AZ61 sheets were determined by uniaxial tensile tests of miniaturized samples with a gauge length of 10 mm and a cross-section of 1.6 × 1.2 mm^2^, which were conducted on a Zwick/Roell (Ulm, Germany) Z005 testing machine equipped with a 1 kN load cell. The tensile test methodology has been described more in detail in [32,33]. The miniaturized samples were machined from the sheets with their tensile direction parallel to the RD (0°), TD (90°), and at an angle of 45° to the RD. Each direction was represented by three test specimens. Tensile experiments were performed at an initial strain rate of 10^−3^ s^−1^ using a Digital Image Correlation (DIC) method for strain measurement [34]. A strain hardening exponent (*n*) was also calculated based on a relation between true stress (*σ*), true strain (*ε*), and strength coefficient (*K*) within a uniform deformation regime, which can be presented as follows [35]:

(1) $$\sigma =K\varepsilon^{n}$$

The mechanical characteristics of sheets was analyzed based on the average value $\bar{X}$ of each parameter, i.e., 0.2% offset yield strength—YS, ultimate tensile strength—UTS, uniform elongation—A_u_, elongation to failure—A, strain hardening exponent—*n*, calculated using the following formula [36]:

(2) $$\bar{X}=\left(X_{0}+2X_{45}+X_{90}\right)/4$$

where *X* denotes to YS, UTS, A_u_, A, or *n* value, and the subscript indicates an angle to the RD.

## 3. Results and Discussion

### 3.1. Microstructure

**Figure 1:**
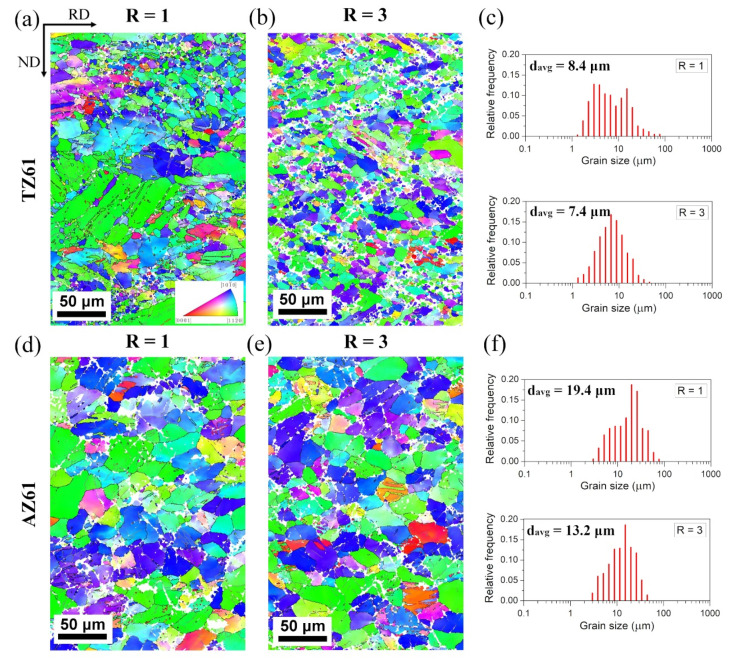
EBSD inverse pole figure (IPF) maps and grain size distributions of the as-rolled (**a**–**c**) TZ61 and (**d**–**f**) AZ61 sheets processed at speed ratio of: (**a**,**c**) R = 1 and (**b**,**d**) R = 3.

**Figure 2:**
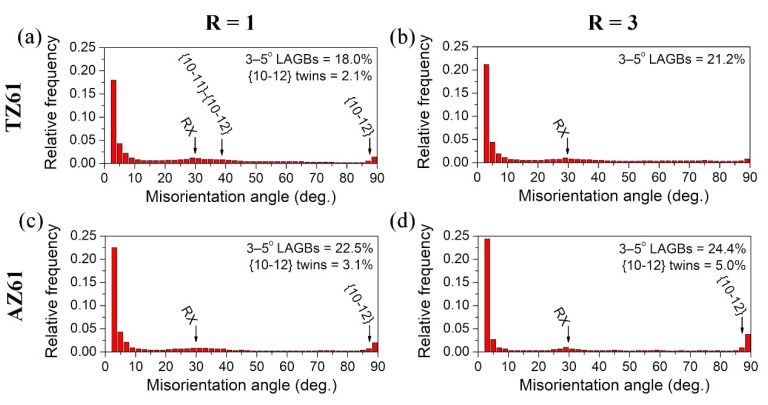
The misorientation angle distributions of the as-rolled (**a**,**b**) TZ61 and (**c**,**d**) AZ61 sheets processed at speed ratio of: (**a**,**c**) R = 1 and (**b**,**d**) R = 3.

EBSD inverse pole figure (IPF) maps of the as-rolled TZ61 and AZ61 sheets are presented in Figure 1, whereas the misorientation angle distributions are shown in Figure 2. The microstructure of the conventionally rolled TZ61 sheet was composed of a high fraction of small recrystallized grains with a size of 2–5 µm and relatively coarse grains with a size of 20–100 µm with a number of twins inside them (Figure 1a). A slightly higher fraction of grain boundaries (GBs) with a misorientation angles of around 30° (Figure 2a) corresponded to newly-formed recrystallized (RX) grains [37,38] while weak peaks at about 86° and 38° indicated the activity of {10 − 12}<10 − 11> extension twinning and {10 − 11}-{10 − 12} double twinning, respectively [39,40]. The higher accumulated strain during DSR processing induced higher grain refinement of TZ61 sheet (Figure 1b). TZ61 alloy processed at R = 3 exhibited a relatively homogeneous microstructure with a reduced average grain size of 7.4 µm (in comparison to 8.4 µm for R = 1). The bimodal grain size distribution observed for R = 1 has vanished for the DSR-processed (R = 3) TZ61 sheet (Figure 1c). Besides, the misorientation angle distribution for DSR-processed sheet (Figure 2b) exhibited slightly enlarged fraction of LAGBs with the misorientation angle less than 5°, whereas the weak peaks indicating twin boundaries has almost disappeared. The reduced activity of twinning in the DSR-processed TZ61 sheet resulted from the smaller grain size. It is known that stresses required to activate twinning becomes larger with grain size refinement [41,42], while below a certain critical value (e.g., 2.7 µm for pure Mg [43]) it can be completely inhibited.

The microstructure of both as-rolled AZ61 sheets comprised of grains with a size of 10–30 µm showing a relatively uniform grain size distributions (Figure 1d–f). The average grain size for conventionally (R = 1) as well as asymmetrically (R = 3) rolled AZ61 sheets were larger than for TZ61 sheets, i.e., 19.4 and 13.2 µm for R = 1 and R = 3, respectively. The misorientation angle distributions exhibited the dominance of LAGBs with a misorientation angle below 5° (Figure 2c,d), which suggests the presence of a high density of dislocations and low-angle dislocation boundaries. Moreover, the DSR process of AZ61 alloy resulted in a greater accumulation of both LAGBs < 5° and {10 − 12}<10 − 11> extension twins with a misorientation angle of about 86°.

**Figure 3:**
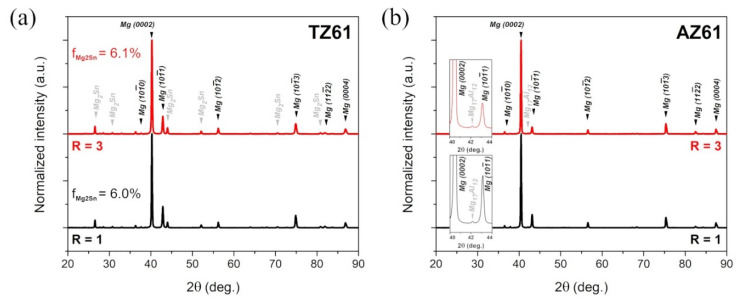
XRD patterns of as-rolled (**a**) TZ61 and (**b**) AZ61 sheets processed at speed ratio of R = 1 and 3.

It should be noted that Mg-Sn-based alloys are a heat-treatable Mg alloys [14,44,45] and thus, the annealing and plastic deformation at high temperature results in the formation of Mg_2_Sn precipitates. Figure 3 presents the XRD patterns for the as-rolled TZ61 and AZ61 sheets. The peaks coming both from Mg lattice as well as Mg_2_Sn phase (peaks at 2θ values of around 26.5, 30.7, 43.9, 52.0°, etc. [46,47]) were observed for TZ61 sheets. The semi-quantitative analysis showed that the volume fraction of Mg_2_Sn phase (f_Mg_2_Sn_) was around 6.0–6.1% (Figure 3a). In the case of AZ61 sheets, a very weak peak of Mg_17_Al_12_ phase (around 2θ = 42.2°) was distinguished, but its small intensity did not allow for the precise determination of Mg_17_Al_12_ volume fraction, which was under the detection threshold, i.e., below 2%. Nevertheless, the significantly higher fraction of second phase precipitates in the TZ61 alloy seems to effectively limit the grain growth during intermediate annealing and high temperature deformation that led to lower average grain size for TZ61 as compared to AZ61 sheets.

**Figure 4:**
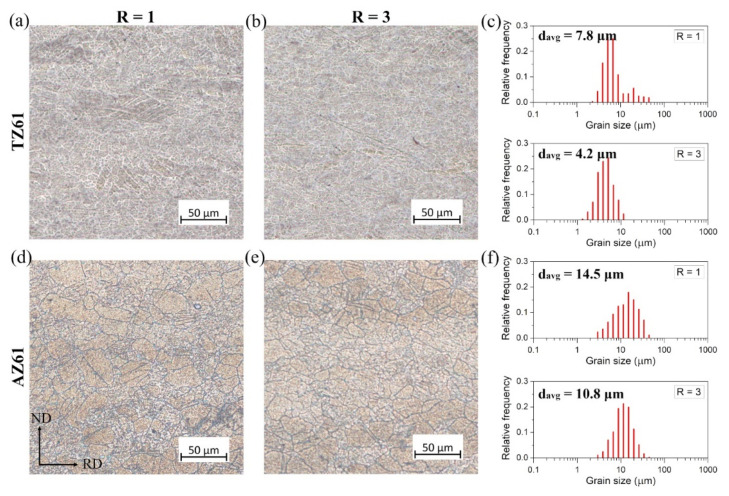
LM images of the microstructure and grain size distributions of the annealed (**a**–**c**) TZ61 and (**d**–**f**) AZ61 sheets processed at speed ratio of: (**a**,**c**) R = 1 and (**b**,**d**) R = 3.

The annealing process at 300 °C of the TZ61 and AZ61 sheets resulted mostly in the formation of homogeneous recrystallized microstructures with equiaxed grains as presented in Figure 4. The only exception was the conventionally rolled TZ61 sheet for which some elongated grains with a size of 20–50 µm (a remnant of coarse grains with twins noted in the as-rolled state) were still visible. For both investigated alloys, the reduced grain size was obtained for DSR-processed sheets that results from a higher accumulated plastic strain energy as well as smaller grain size after DSR. What is more important, the significantly lower average grain sizes were estimated for TZ61 sheets (7.8 and 4.2 µm for R = 1 and 3, respectively) in comparison to AZ61 sheets (14.5 and 10.8 µm for R = 1 and 3, respectively). On the one hand, the higher fraction of twin boundaries and LAGBs < 5° (i.e., higher density of dislocations and dislocation boundaries) in the AZ61 sheets (Figure 2) may suggest their higher propensity for new RX grains formation. However, on the other hand, a higher number of Mg_2_Sn phase precipitates in the TZ61 sheets hinders grain growth more effectively than Mg_17_Al_12_ that leads to a pronounced grain refinement in the TZ61 sheets.

### 3.2. Texture

**Figure 5:**
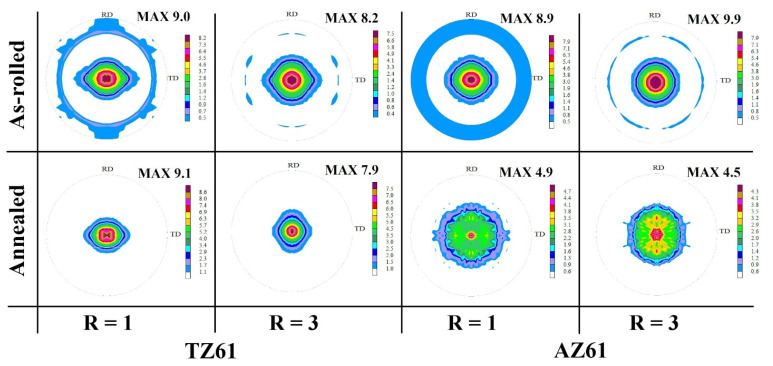
The (0002) pole figures of the as-rolled and annealed TZ61 and AZ61 sheets processed at R = 1 and 3.

**Figure 6:**
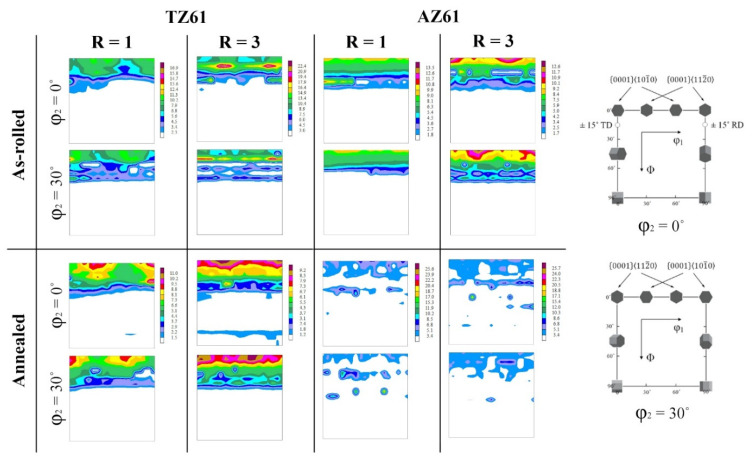
The ODFs (ϕ_2_ = 0° and 30° sections) of as-rolled and annealed TZ61 and AZ61 sheets processed at R = 1 and 3.

The recalculated (0002) pole figures of the as-rolled TZ61 and AZ61 sheets are presented in Figure 5, while the main texture components has been visualized in the form of ϕ_2_ = 0° and 30° ODF sections in Figure 6. All analyzed sheets exhibited a relatively strong basal texture, which is normally observed for the as-rolled Mg alloys [2,4]. The intensity of basal texture decreased after DSR processing from 9.0 to 8.2 MRD (i.e., multiples of random distribution) for TZ61 alloy and from 9.9 to 8.9 MRD for AZ61 alloy. As we can see, the higher basal texture intensity was noted for AZ61 sheets processed at both speed ratios. It has been also shown that a slight splitting of basal poles has occurred for TZ61 sheets, while there was no such phenomenon in the case of AZ61 sheets. The (0002) poles in the TZ61 sheets were split to the TD and RD for R = 1 and R = 3, respectively. The tilting of basal poles was easier to observe on the ODFs. It should be pointed out that tilting towards TD and RD can be recognized by a movement of (ϕ_1_ = 0°, Φ = 0°, ϕ_2_ = 0°) and (90°, Φ, 0°) texture components to the higher value of the Φ angle, respectively [48]. For the conventionally rolled TZ61 sheet, the strongest texture component at (0°, 10°, 30°) suggested that the TD spreading occurred, whereas for DSR-processed TZ61 sheet (~90°, 10°, 0°) peak indicated on the RD tilting. Both AZ61 sheets exhibited a strong single-peak basal texture, which is common for Mg-Al-Zn alloys [1,2,4]. The basal texture spreading has been observed so far mainly for Mg-RE [49], Mg-Zn [50], or Mg-Li [51] alloys, but our latest study [27] as well as Verma et al. [52] have shown that it is possible to obtain basal texture splitting in the Mg-Sn-based alloys as well. The reason of the RD and TD spreading seems to be the activation of pyramidal <c + a> and prismatic <a> slip, respectively [51,53]. The pyramidal <c + a> slip is of particular importance for Mg alloys because it allows to compensate plastic deformation in the c-axis of Mg hexagonal lattice and contributes to increasing Mg sheets formability [42,54]. Finally, the ODFs clearly showed that {0001}<11 − 20> texture component dominated in the TZ61 sheets with a four intensity peaks visible at around (30°, Φ, 0°), (90°, Φ, 0°), (0°, Φ, 30°), and (60°, Φ, 30°), which were slightly shifted in the ϕ_1_ angle from the ideal position. The main texture component in the AZ61 sheets was {0001}<10 − 10>, which is mostly observed for the Mg-Al-Zn alloys [48,55].

After annealing at 300 °C, the TZ61 sheets still exhibited the basal texture with a similar intensity to their as-rolled counterparts (i.e., 9.1 and 7.9 MRD for R = 1 and R = 3, respectively), although, the RD and TD splitting has been blurred and the {0001}<11 − 20> texture component has been strengthened with its intensity peaks at ideal positions of (30°, 0°, 0°), (90°, 0°, 0°), (0°, 0°, 30°), and (60°, 0°, 30°). In turn, a significant basal texture spreading has been observed for the AZ61 sheets after annealing process with drastically lowered intensity down to 4.9 and 4.5 MRD for R = 1 and R = 3, respectively.

### 3.3. Mechanical Properties

**Figure 7:**
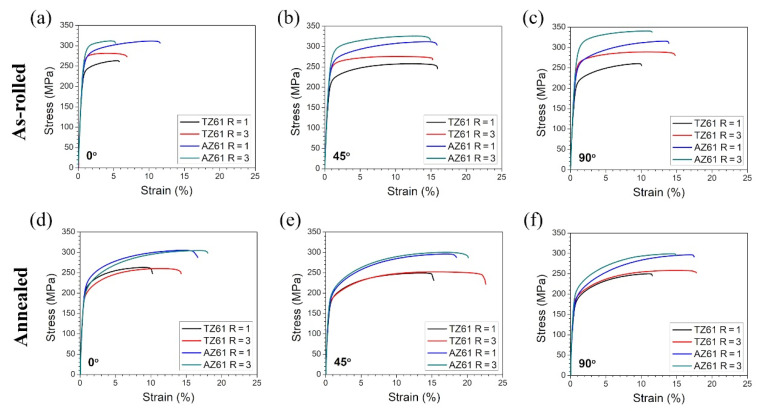
Stress–strain curves for the (**a**–**c**) as-rolled and (**d**–**f**) annealed TZ61 and AZ61 sheets processed at speed ratio R = 1 and 3 at different angles to the RD: (**a**,**d**) 0°, (**b**,**e**) 45°, and (**c**,**f**) 90°.

**Figure 8:**
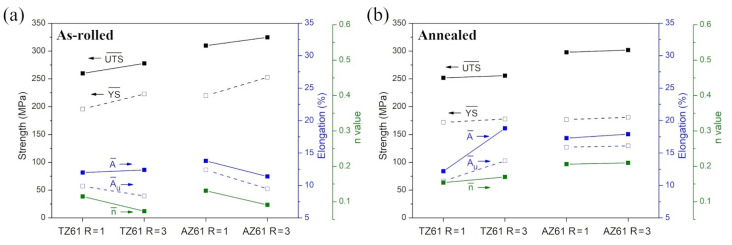
The average values of mechanical properties of the (**a**) as-rolled and (**b**) annealed TZ61 and AZ61 sheets processed at speed ratio R = 1 and 3.

The stress–strain curves obtained for tensile samples cut at different angles to the RD from the TZ61 and AZ61 sheets are presented in Figure 7, while all calculated mechanical parameters are summarized in Table 1 and Figure 8. The results for the as-rolled TZ61 and AZ61 sheets showed that the average values of YS and UTS were enhanced by DSR processing at the expense of the reduced uniform elongation and strain hardening exponent. It should be stated that all sheets exhibited a significant anisotropy of strength and plasticity (i.e., higher YS and UTS with lowered elongation were noted at 0° as compared to 90°) and the estimated *n* values for TZ61 and AZ61 sheets were close to those obtained for the common AZ31 (*n* = 0.09–0.16 [23]) or Mg-6Sn (*n* = 0.10–0.12 [27]) alloys processed by DSR. The enhanced strength of asymmetrically rolled sheets was a result of their higher grain refinement and dislocation density (represented by higher 3–5° LAGBs fraction in Figure 2). Despite the lowered basal texture intensity (Figure 5), DSR processing reduced the ability to uniform plastic deformation of both investigated alloys due to increased number of dislocations/LAGBs and twin boundaries (Figure 2). Moreover, for both speed ratios the strength and plasticity of the as-rolled AZ61 sheets was higher in comparison to their TZ61 counterparts. AZ61 sheets processed at R = 1 and R = 3 exhibited higher YS and UTS by around 30 and 50 MPa, respectively. On the one side, the average grain size in the as-rolled TZ61 sheets was significantly lower than for AZ61 (Figure 1) and thus, the grain boundary strengthening should be greater in the TZ61 sheets. On the other side, the AZ61 sheets showed higher 3–5° LAGBs fraction (Figure 2), which suggests their higher strengthening by dislocations. Nevertheless, the main factor affecting the strength difference in the investigated Mg alloys seems to be a solid solution strengthening. TZ61 alloy is hardened by Mg_2_Sn phase precipitates, but their hardening effect is not so effective as in the case of Al alloys [13,14]. The solid solution strengthening mechanism is controlled by an alloying element content and a mismatch of atomic radius of additive and matrix atoms [2,4]. The higher difference between Mg and Al atoms (in comparison to Mg and Sn) [2] and higher content of Al atoms in Mg solid solution (Sn tends to create Mg_2_Sn precipitates) indicate that the solid solution strengthening effect by Al is more effective in contributing to enhanced YS and UTS of AZ61 sheets as compared to TZ61.

The annealing process at 300 °C caused a decrease of YS and UTS of the rolled TZ61 and AZ61 sheets. The average YS was lowered to 170–180 MPa for both analyzed alloys, while the annealed AZ61 sheets still showed higher average UTS (~300 MPa) as compared to TZ61 sheets (~255 MPa). Since the higher *n* values are an indicator of enhanced ability to uniform plastic deformation, the uniform elongation values tend to grow with higher strain hardening exponents. It should be noted that the greater A_u_ and *n* values were achieved after DSR processing as well as for AZ61 sheets. The enhancement of plasticity after DSR processing can be mainly attributed to the reduced grain size (Figure 4) and lowered basal texture intensity (Figure 5) observed for both analyzed Mg alloys. In turn, the increased uniform elongation of AZ61 alloy in comparison to TZ61 resulted mostly from its weakened texture intensity (Figure 5) and greater ability to uniform plastic deformation (higher *n* values in Table 1). TZ61 sheets contained a much higher amount of Mg_2_Sn precipitates (Figure 3), which restricted their uniform deformation and disabled such a strong strain hardening as in the case of AZ61 sheets. Nonetheless, it should be pointed out that TZ61 alloy was more prone to effective grain refinement (Figure 1) and enhancement of formability (Table 1) by DSR processing. It was possible to obtain even higher elongation to failure for the DSR-processed TZ61 sheet (A = 18.8%) as compared to AZ61 sheet (A = 17.9%), despite of its much higher texture intensity (7.9 and 4.5 MRD for TZ61 and AZ61, respectively). Therefore, further improvement of formability of TZ61 sheets should be addressed to a proper texture modification. Finally, it should be also mentioned that Mg-Sn-based alloys show a much greater potential for high-temperature applications due to the formation of Mg_2_Sn precipitates, which are more thermally stable than Mg_17_Al_12_ precipitates in Mg-Al-Zn alloys [12].

## 4. Conclusions

This work presented the comparison of microstructure, texture, and mechanical properties of the newly developed TZ61 (Mg-6Sn-1Zn) alloy with the commercially available AZ61 (Mg-6Al-1Zn) alloy processed by conventional symmetric rolling and differential speed rolling (DSR). The main conclusions were found as follows:

− The reduced grain size in the TZ61 and AZ61 sheets was obtained by DSR processing in comparison to the conventional rolling. A high number of Mg_2_Sn precipitates in the TZ61 sheets effectively hindered grain growth during intermediate annealing and high temperature deformation led to their smaller grain size as compared to AZ61 sheets.
− DSR processing lowered also the basal texture intensity in the TZ61 and AZ61 sheets. The as-rolled TZ61 sheets exhibited the basal poles splitting to TD or RD, while a single-peak basal texture was observed in the case of AZ61 sheets. The main texture component for TZ61 and AZ61 sheets was {0001}<11 − 20> and {0001}<10 − 10>, respectively.
− The plasticity of the annealed TZ61 and AZ61 sheets was enhanced by DSR processing. AZ61 sheets showed higher uniform elongation and UTS values (as compared to TZ61 ones), which has been attributed to their significantly lowered texture intensity after annealing and greater ability to strain hardening.
− It should be pointed out that TZ61 alloy was more prone to effective grain refinement and enhancement of formability by DSR processing. It was possible to obtain even higher elongation to failure for the DSR-processed TZ61 sheet (A = 18.8%) as compared to AZ61 sheet (A = 17.9%), despite its much higher texture intensity.

## Figures and Tables

**Table 1:** Mechanical properties ($\bar{YS}$—yield strength, $\bar{UTS}$—ultimate tensile strength, $\bar{A_{u}}$—uniform elongation, $\bar{A}$—elongation to failure, *n*—strain hardening exponent) of the as-rolled and annealed TZ61 and AZ61 sheets processed with different speed ratio.

**State**	**Alloy**	Speed Ratio	Direction	YS (MPa)	UTS (MPa)	A_u_ (%)	A (%)	*n*	$\bar{YS}$ (MPa)	$\bar{UTS}$ (MPa)	$\bar{A_{u}}$ (%)	$\bar{A}$ (%)	$\bar{n}$
As-rolled	TZ61	R = 1	0°	211 ± 7	265 ± 7	4.9 ± 2.1	6.0 ± 3.1	0.081 ± 0.004	196	260	9.9	12.0	0.115
			45°	190 ± 5	258 ± 3	12.6 ± 0.8	15.9 ± 0.4	0.122 ± 0.005					
			90°	184 ± 6	258 ± 7	9.6 ± 2.4	10.3 ± 3.1	0.134 ± 0.001					
		R = 3	0°	234 ± 7	278 ± 5	3.6 ± 0.8	6.1 ± 1.7	0.045 ± 0.003	223	278	8.4	12.4	0.073
			45°	215 ± 3	274 ± 3	9.5 ± 0.3	15.0 ± 1.5	0.076 ± 0.001					
			90°	226 ± 8	285 ± 5	10.8 ± 0.5	13.5 ± 0.9	0.096 ± 0.007					
	AZ61	R = 1	0°	240 ± 5	312 ± 2	9.7 ± 0.5	11.0 ± 0.7	0.103 ± 0.002	220	310	12.4	13.8	0.131
			45°	213 ± 1	308 ± 3	13.6 ± 0.3	15.3 ± 0.6	0.136 ± 0.006					
			90°	215 ± 3	313 ± 3	12.7 ± 1.7	13.7 ± 1.7	0.147 ± 0.004					
		R = 3	0°	258 ± 5	312 ± 4	5.2 ± 1.8	6.2 ± 3.1	0.066 ± 0.006	253	325	9.5	11.4	0.091
			45°	248 ± 2	326 ± 4	11.6 ± 0.7	14.4 ± 0.9	0.100 ± 0.005					
			90°	258 ± 1	336 ± 5	9.6 ± 2.0	10.5 ± 2.5	0.098 ± 0.005					
Annealed	TZ61	R = 1	0°	199 ± 6	262 ± 3	8.3 ± 1.0	9.8 ± 1.9	0.130 ± 0.001	172	252	10.7	12.2	0.154
			45°	166 ± 2	249 ± 1	12.1 ± 1.1	14.1 ± 1.7	0.156 ± 0.001					
			90°	156 ± 6	248 ± 3	10.2 ± 1.4	10.7 ± 1.7	0.173 ± 0.006					
		R = 3	0°	188 ± 2	261 ± 1	11.4 ± 0.7	14.5 ± 1.1	0.155 ± 0.003	178	256	13.8	18.8	0.170
			45°	174 ± 3	252 ± 1	14.7 ± 0.4	21.8 ± 0.5	0.171 ± 0.004					
			90°	175 ± 5	257 ± 1	14.4 ± 0.3	17.2 ± 1.8	0.182 ± 0.009					
	AZ61	R = 1	0°	197 ± 4	304 ± 2	14.2 ± 0.1	16.2 ± 0.1	0.175 ± 0.005	177	298	15.9	17.3	0.206
			45°	171 ± 7	295 ± 3	16.7 ± 0.6	18.4 ± 0.5	0.210 ± 0.002					
			90°	168 ± 3	296 ± 1	15.8 ± 0.5	16.4 ± 0.8	0.230 ± 0.008					
		R = 3	0°	182 ± 3	303 ± 2	16.1 ± 0.6	17.7 ± 1.1	0.200 ± 0.006	181	302	16.1	17.9	0.210
			45°	178 ± 1	299 ± 2	16.3 ± 0.1	19.0 ± 1.0	0.218 ± 0.003					
			90°	187 ± 5	306 ± 3	15.5 ± 0.5	15.9 ± 0.5	0.206 ± 0.007					

## Data Availability

The data presented in this study are available on request from the corresponding author.
